# Development and validation of the Character Strengths Test 24 (CST24): a brief measure of 24 character strengths

**DOI:** 10.1186/s40359-023-01280-6

**Published:** 2023-08-18

**Authors:** Satoshi Shimai, Yu Urata

**Affiliations:** 1 Department of Psychological Sciences, Kansai University of Welfare Sciences, Kahiwara, Osaka, Japan; 2 Center for Student Success Research and Practice, Osaka University, Toyonaka, Osaka, Japan

**Keywords:** Character strengths, Test development, Validity, Reliability, Positive psychology

## Abstract

**Background:**

The present study aimed to develop and validate the Character Strengths Test 24 (CST24), a simple scale consisting of 24 character strengths represented by one concept word and one sentence each. Three studies were conducted to examine the validation and utility of the CST24 for future research.

**Methods:**

Three internet-based surveys were conducted in Japan to investigate the psychometric properties of the CST24. Study 1 comprised 846 adults and focused on test development, including the evaluation of well-being and strengths scales, as well as retest reliability. Study 2 involved 1137 high school students and 1101 college undergraduates, aiming to investigate the factor structure of the CST24 and reaffirm its validity by utilizing happiness and meaning in life scales. In Study 3, 524 working adults participated to explore the scale's potential for future research. This study incorporated various psychological scales, such as value orientation, moral foundation, and work-related scales, to assess the utility of the CST24.

**Results:**

In Study 1, the CST24 items exhibited a well-distributed response pattern, demonstrating favorable retest reliability and internal consistency. Significant positive correlations were found between the CST24 items and measures of subjective well-being, meaning in life, positive self-compassion, and knowledge or utilization of strengths. Study 2 confirmed the stability of the factor structure across diverse sample groups, consistent with prior studies utilizing larger-scale measures. Study 3 identified both core and peripheral strengths, highlighting specific strengths that made substantial contributions to well-being, value orientation, moral foundation, sense of mission, and work-related indices through multiple regression analysis.

**Conclusion:**

The findings support the reliability and validity of the CST24 as a concise assessment tool for measuring 24 character strengths. Its potential utility for screening and exploratory research warrants attention in future studies, enhancing our understanding of the role of character strengths in various domains.

**Supplementary Information:**

The online version contains supplementary material available at 10.1186/s40359-023-01280-6.

## Introduction

The study of character strengths aims to establish a comprehensive inventory of positive psychological functions in humans. Peterson and Seligman [1] proposed ten criteria for the selection of these strengths, including distinctiveness and independence from other strengths. Additionally, these strengths should contribute to personal well-being, enhance interpersonal relationships, and align with societal and cultural traditions [2]. The Values-in-Action, Inventory of Strengths (VIA-IS), a 240-item scale developed by Peterson, serves as the standard measure for assessing character strengths [3]. While the VIA-IS has been used successfully in numerous studies across different countries [4, 5], there are practical limitations, particularly in the Japanese context.

In Japan, a Japanese version of the VIA-IS has been developed [6], but its use in research has been limited due to practical difficulties. Firstly, the scale's large number of items makes it challenging to integrate with other measures or incorporate into broader research projects. Secondly, the VIA Research Institute holds the copyright for the Japanese version, restricting access to researchers. The limited availability of Japanese VIA-IS data has hindered character strengths research in Japan.

Peterson, Park & Castro [7] developed the Global Assessment Tool (GAT), which includes a 24-item Brief Strengths Test (BST) as one of its components. The test item is, for example, in Creativity, " Think of actual situations in which you had the opportunity to do something that was novel or innovative. How frequently did you show CREATIVITY or INGENUITY in these situations?” The remaining items in the scale follow a consistent format, with minor modifications involving an underlined explanation and the substitution of strength names [8]. Although the BST is included in the GAT used by the U.S. Army, there is a lack of scientific studies examining its validity as a standalone scale.

Vanhove, Harms & DeSimone [9] sought to establish the validity of the BST in the general population. They conducted factor analyses and identified a reduced version of the scale, excluding strengths such as honesty/integrity, self-control, and spirituality. However, the rationale for excluding these strengths was not clearly explained, limiting our understanding of human strengths. Another study by Vie, Scheier, Lester & Seligman [10] proposed excluding different strengths (courage, diligence, enthusiasm, aesthetics, social intelligence, and spirituality) based on a large analysis of military data, resulting in a stable factor structure. Again, the reasoning behind these exclusions remains unexplained.

In contrast, Ruch, Martínez-Martí, Proyer, & Harzer [11] developed a shorter scale, the Character Strengths Rating Form (CSRF), to overcome practical challenges in implementing longer scales concurrently with other research measures and for longitudinal studies. Similar to the BST, the CSRF consists of 24 items, with each strength followed by multiple explanatory sentences. For example, the first item is "Creativity (originality, ingenuity):" followed by “Creative people have a highly developed thinking about novel and productive ways to solve problems and often have creative and original ideas. They do not content themselves with conventional solutions if there are better solutions”.　The CSRF demonstrated validity through its resemblance to VIA responses, a five-factor structure derived from exploratory factor analysis, and positive associations between the factors and life satisfaction.

In this study, we aim to develop a practical and time-efficient scale, the Character Strengths Test 24 (CST24), similar to previous attempts such as the CSRF. While unaware of the CSRF during the initial development phase, we now acknowledge this as the second attempt to create a shortened self-administered character strengths scale. Our objectives include examining the validity, reliability, and distinguishing features of the CST24 compared to the CSRF, as well as exploring the scale's utility through exploratory analyses.

The CST24 format follows that of the CSRF, presenting the name of each strength followed by an explanatory sentence. However, unlike the CSRF, the CST24 provides a single-word name for each strength and does not offer a paraphrase. Instead, the sentence aims to encompass the breadth of meaning associated with the paraphrase. For example, “Creativity: I come up with new ways of seeing and thinking, and use them to solve problems in unique ways”. The sentences adopt a first-person perspective (using "I"), similar to the VIA-IS. This format enhances precision in judgment due to the succinctness of the sentence and the clear identification of the self as the subject. The sentence development process involved the first author, who previously developed the Japanese version of the VIA-IS [6], and incorporated feedback from psychology experts and preliminary surveys with diverse age groups. The final version was refined based on participant impressions and response patterns. Response options utilize a 7-point Likert scale, with each option expressing the degree to which it applies to the individual.

## Study 1

Study 1 aims to investigate the validity and reliability of the Character Strengths Test-24 (CST24) by examining its response characteristics, retest reliability, convergent validity, construct validity, discriminant validity, and factor structure. This study also seeks to explore correlations between CST24 items and subjective happiness [12], presence of meaning in life [13], strength knowledge, utilization scales [14], and self-compassion [15]. Additionally, the factor structure of the CST24 is examined using confirmatory factor analysis using two- to six factor models [16].

## Methods

### Participants

A total of 846 Japanese adults (401 males and 445 females) out of 1185 registered with an Online survey agency participated in the study. The mean age of the participants was 45.48 ± 13.90 years (ranging from 20 to 69). The marital status of the participants included 34.9% never married, 57.9% married, and 7.2% bereaved or separated. Regarding educational and occupational backgrounds, 61.6% had a university or college degree or higher as their last education, and 52.2% of the participants had full-time jobs or were self-employed. Additionally, 12.6% worked part-time, 18.2% were housewives, and 16.9% were either unemployed or students.

### Procedure

An Online survey agency conducted the survey among Japanese adults. Participants were provided with the purpose and informed consent of the study on-screen and were asked to respond to the survey after understanding and agreeing to participate. To ensure response accuracy, participants were prompted to change their answers if the same response was selected for all 24 CST items. Moreover, 339 respondents (28.6% of 1185 participants) who consistently selected the same response for both the Subjective Happiness Scale and the Meaning in Life Questionnaire, which contained reversal items, were excluded from data analysis. To examine retest reliability, 424 participants (212 males and 212 females) were asked to respond to the CST24 items again after a 30-day interval, and the correlation between the two sets of data was analyzed. The survey company ensured the anonymity of participants, providing the researchers with de-identified data. All statistical analyses were performed using SPSS 28.0 and AMOS 24.0.

### Measures

#### Character Strengths Test-24 (CST24)

The CST24 measures 24 character strengths through a single sentence description for each strength. These sentences were developed based on the Japanese version of the Values-in-Action Inventory of Strengths (VIA-IS) [6], and no reversal items were included. Participants rated their agreement with each sentence using a 7-point Likert scale ranging from "not at all applicable [1]" to "very applicable [7]." Table 1 presents all the strength items. The development of the CST24 involved careful creation in Japanese by the first Japanese author, who specializes in positive psychology and has previously developed the Japanese version of the VIA-IS. The wording was reviewed by colleagues specialized in psychology. The sentences presented here are the English translations of the CST24 items.

**Table 1:**
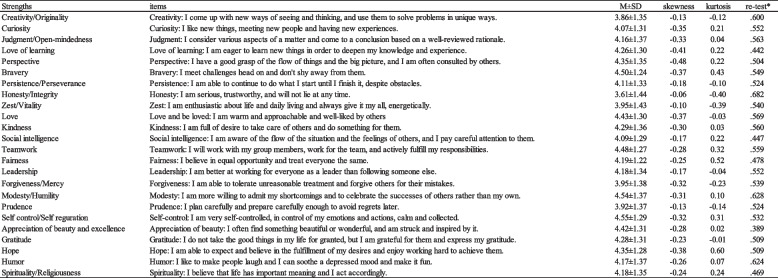
CST24 items and mean and descriptive statistics (*n* = 846) and re-test reliability (*n* = 424)

^*^Correlation coefficients of retests (*n* = 424, *p* < .001 for all)

#### Subjective Happiness Scale (SHS) and Meaning in Life Questionnaire (MLQ)

The SHS, developed by Lyubomirsky and Lepper [12], measures subjective well-being. Participants respond to a 7-item Likert scale to indicate their level of happiness [17]. The Japanese SHS has shown a negative correlation with mental illness and to be reliable and valid [18]. The MLQ, developed by Steger, Frazier, Oishi, and Kaler [19], consists of two subscales: presence of meaning in life and search for meaning in life. The subscale score is calculated by summing the responses to five items. The Japanese version of the MLQ has demonstrated both reliability and validity in academic research. Specifically, the "presence score," which measures one's comprehension of life's significance, exhibits a robust correlation with the well-being index [20].

#### Strength Knowledge and Use Scale (SKS and SUS) and Self-Compassion Scale (SCS)

The SKS and SUS were developed by Govindji and Linley [14] to measure awareness of one's strengths and the extent to which individuals utilize their strengths on a daily basis. Responses are provided on a 5-point scale ranging from "not at all (1 point)" to "very well (5 points)" The Japanese version of the SCS short form, including subscales, has shown strong validity and reliability in previous studies [21, 22]. It encompasses both positive and negative aspects of self-compassion [23]. Participants rate their agreement with various statements on a 5-point scale ranging from "almost never [1]" to "almost always [5]".

Before responding to the psychological measures mentioned above, participants were asked to provide demographic information such as gender, age, marital status, educational background, occupation, and income.

## Results and discussion

### Response distribution

Table 1 presents the response distribution statistics, including correlation coefficients, mean ± SD, kurtosis, skewness, and retest reliability for the 24 item statements of the CST24. The CST24 is a self-assessment measure using a 7-point Likert scale (ranging from 1 to 7). The mean scores ranged from 3.61 for Honesty to 4.55 for Self-control, with an overall distribution centered around the midpoint of the scale [4], indicating no ceiling or floor effects. Skewness values ranged from -0.06 for Honesty to -0.048 for Perspective, while kurtosis values ranged from -0.32 for Persistence to 0.60 for Hope. Significant positive correlations were observed among all strength scores, with the smallest correlation observed between Curiosity and Prudence (*r* = 0.215), and the largest correlation observed between Appreciation of beauty and excellence and Gratitude (*r* = 0.671). The Cronbach's alpha coefficient for all 24 items was 0.953, and deleting any item did not improve the coefficient. The retest reliability coefficients over a 30-day interval indicated that 19 out of 24 strengths demonstrated adequate reliability with coefficients of 0.5 or higher.

### Construct validity

Table 2 displays the correlation coefficients between each strength score and measures of subjective well-being (SHS) and presence of meaning in life (MLQ-p). All correlations were significant and positive. The largest correlations with SHS were observed for Hope (0.526), Zest (0.467), and Spirituality (0.451), while the smallest correlation was found for Prudence (0.223). Regarding MLQ-p, the highest correlations were observed for Zest (0.580), Spirituality (0.560), and Hope (0.463), whereas the lowest correlation was observed for Prudence (0.250). Furthermore, the strength scores were positively correlated with the knowledge and utilization of strengths (SKS and SUS). Among these correlations, Hope (0.577) and Zest (0.624) displayed the strongest associations, while Prudence showed relatively lower correlations (0.353 and 0.369, respectively), aligning with the results obtained from SHS and MLQ assessments. Additionally, all strengths demonstrated significant positive associations with the Positive Self-Compassion Scale (SC), and conversely, significant negative associations with the Negative Self-Compassion Scale (SC).

**Table 2:**
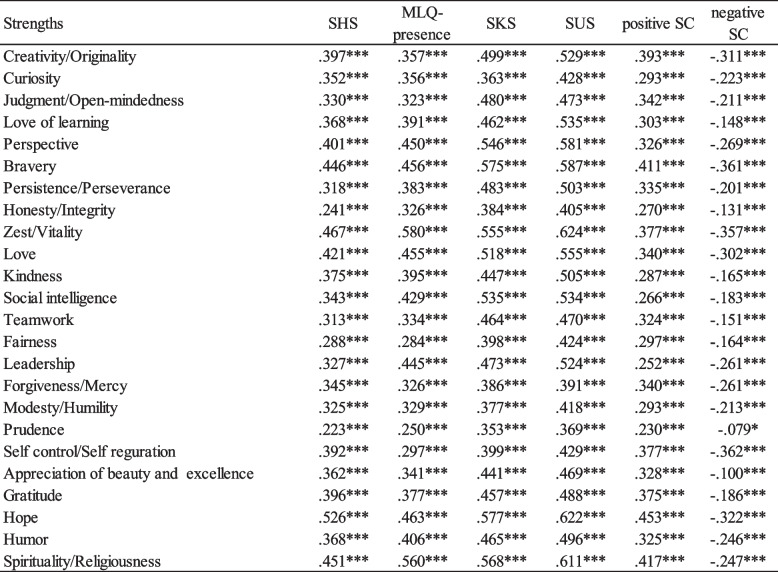
Correlation coefficients of strengths with subjective happiness (SHS), meaning in life (MLQ), knowledge (SKS) and use of strengths (SUS), and positive and negative self-compassion (SC)

^***^p < .001,**p < .01,*p < .05

### Structure examination by confirmatory factor analysis

Confirmatory factor analysis (CFA) was conducted to examine the factor structure of the strengths, following the approach of Vanhove et al. [16], who proposed 2- to 6-factor models. In line with their suggestions, the 23-strength model included three-factor and four-factor models, excluding spirituality in the three-factor model and open-mindedness in the four-factor model, as recommended by Shryack et al. [24]. Additionally, the present study explored the CFA results using all 24 strengths, grouping them into conscientiousness, according to the high factor loadings of these two strengths in their study [24].

Table 3 presents the findings of the seven CFA models, including the two-factor model with a CFI of 0.847 and the six-factor model with the highest fit indices (CFI = 0.888), but not reaching the threshold of 0.900. The RMSEA values for all models were below 1, with the six-factor model showing the lowest value at 0.082. The AIC and BIC values increased when considering the model with 24 strengths compared to the model with 23 strengths. The SRMR values were 0.0549 for the highest two-factor model and 0.0499 for the lowest six-factor model. The CFI and RMSEA values did not exhibit substantial changes across the different models. In summary, among all the factor models examined, the six-factor model displayed the best fit, although the difference in fit indices was not substantial.

**Table 3:**
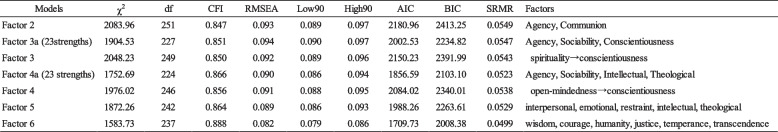
Confirmatory factor analysis model results with CST24 in Study 1

## Study 2

In Study 2, two sample groups were utilized: high school students and freshmen and sophomores of university. The primary objective was to reaffirm the characteristics and validity of each strength score measured by the CST24 and investigate the structure of the CST24 in two distinct age groups using confirmatory factor analysis (CFA), similar to Study 1. Additionally, the study aimed to explore the explanatory power of multiple regression analysis, with subjective happiness and presence of meaning in life as outcome variables and the 24 strengths as predictors.

## Methods

### Participants

The participants consisted of 1098 Japanese high school students (410 males and 688 females) and 1101 Japanese college students (436 males and 665 females) recruited through an Online survey agency. The mean age (± standard deviation) was 17.09 ± 0.81 (ranging from 16 to 18) years for high school students and 19.47 ± 0.70 (ranging from 18 to 22) years for freshmen and sophomores of university. Among all participants in Study 2, only eight university students were married.

### Procedure

An Online survey agency facilitated the survey administration among Japanese high school and university students. Participants were presented with the study's purpose and informed consent on-screen, and those who agreed to participate proceeded to respond to the survey. The survey procedure closely followed that of Study 1, including the selection of responses for all 24 CST items, participants being alerted while responding, and reminders to review and revise their answers.

### Measures

The Character Strengths Test (CST24), a 24-item scale developed in Study 1, was employed. The Subjective Happiness Scale (SHS) and the Meaning in Life Questionnaire—Presence subscale were the same measures used in Study 1.

## Results and discussion

### Response distribution and correlations

Table 4 presents the mean ± standard deviation of the CST24 scores and the correlation coefficients with subjective happiness (SHS) and presence of meaning in life scores (MLQ-p) for the high school and college student groups. Skewness and kurtosis exhibited similar patterns in both groups, consistent with the findings in Study 1. The rankings of strengths were similar to those of Japanese VIA study [6]. The results using the Japanese VIA with university students [6] led to the division of strengths into two groups based on their rankings. A subsequent analysis examined the differences in rankings between the two groups of strengths in this study, revealing significant differences among both high school and university students (*p* < 0.05 for both).

**Table 4:**
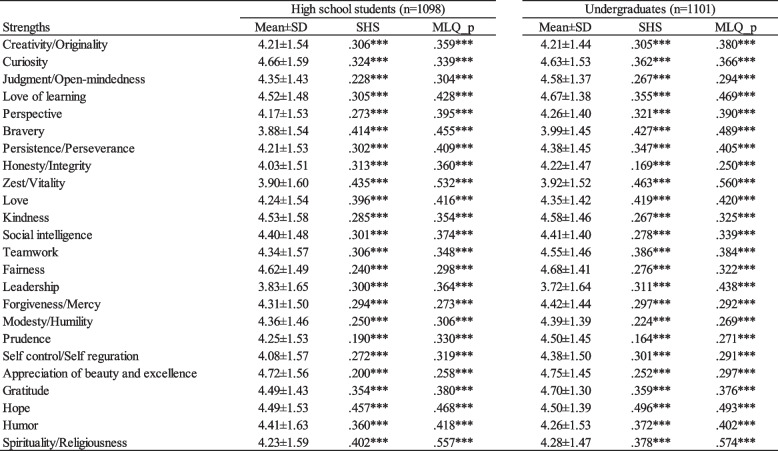
Mean and SD of 24 character strengths and their correlations with happiness (SHS) and meaning in life (MLQ-p) scales among high school and university students

^***^p < .001

Correlational analysis revealed significant positive associations between all strength scores and subjective happiness and presence of meaning in life in both groups. Among high school students, the smallest correlation coefficient with subjective happiness was 0.190 for Prudence, while the largest was 0.457 for Hope. Also, in college students, the smallest coefficient was 0.164 for Prudence, and the largest was 0.496 for Hope. The findings small coefficients for Prudence with SHS were consistent with that in Study1. For the presence of meaning in life, slightly higher correlation coefficients were observed, with a minimum of 0.258 for Appreciation of beauty and excellence and a maximum of 0.557 for Spirituality among high school students, and a minimum of 0.250 for Honesty and a maximum of 0.574 for Spirituality among college students. These findings of correlations were compatible to those of Japanese university student study with VIA-IS [6].

### Validation of structure

Tables 5 and 6 display the results of the CFA examining the two- to six-factor models of the CST24 for high school and college students, respectively. Notably, the results present the models utilizing all 24 strengths. For high school students (Table 5), the two-factor model yielded a CFI of 0.854, while the six-factor model exhibited the highest CFI value of 0.910. The six-factor model demonstrated sufficiently low RMSEA values and SRMR values below 0.05. The four-factor and five-factor models also yielded SRMR values below 0.05, and although the RMSEA values were acceptable, they were comparatively less favorable than the six-factor model. Regarding college students (Table 6), although the six-factor model produced the best fit, the RMSEA values were comparable across the different models. Both the RMSEA and SRMR values were low when compared to those of high school students.

**Table 5:**
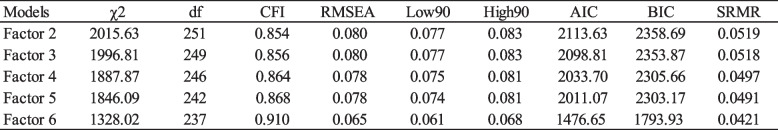
Confirmatory factor analysis model results with CST24 among high school students (n = 1098) in study 2

**Table 6:**
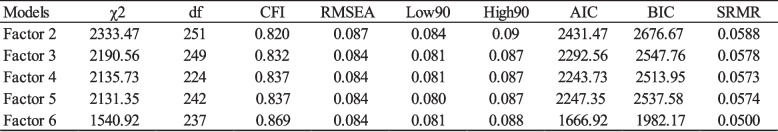
Confirmatory factor analysis model results with CST24 among among freshmen and sophomore student (n = 1101) in Stdy 2

### Multiple regression analysis of happiness and meaning of life

Exploratory multiple regression analyses were conducted separately for high school and college students, with happiness and meaning in life scores as dependent variables and the CST24 as the independent variable. The analyses aimed to determine if the strengths sufficiently accounted for the variance in happiness and meaning in life scores and to identify the specific strengths that significantly contributed to these outcomes. The correlation analysis had previously indicated that all strengths contributed to happiness.

The results, presented in Table 7, reveal that among high school students, the strengths accounted for 31.5% of the variance in subjective happiness and 43.0% in meaning in life. For college students, the corresponding values were 37.2% and 48.0%, respectively, suggesting a substantial contribution of strengths in both groups. Also it was found that the variance for the meaning in life was higher than the variance for subjective happiness in all three groups.

**Table 7:**
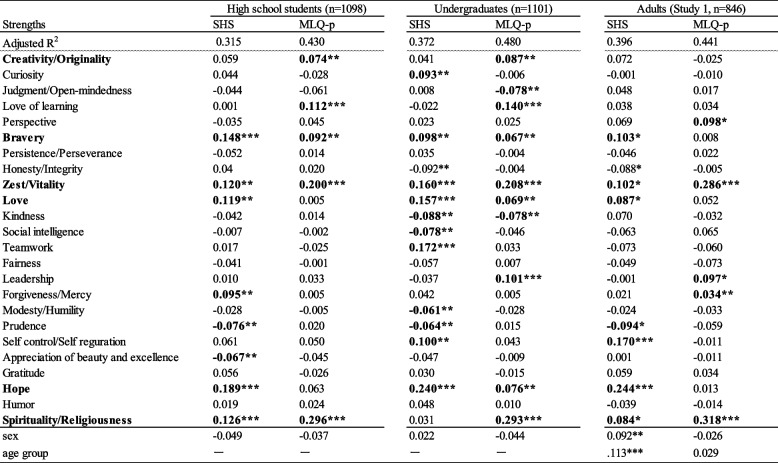
Mean and SD of 24 character strengths and their correlations with happiness (SHS) and meaning in life (MLQ-p) scales among high school and university students

^*^p < .05, **p < .01,***p < .001

Notably, among high school students, the strengths that significantly contributed to happiness were Hope (0.189), Bravery (0.148), Spirituality (0.126), Zest (0.120), and Love (0.119), in descending order. Among college students, Hope took the leading position (0.240), followed by Teamwork (0.172), Zest (0.160), Love (0.157), and Self-regulation (0.100). Additionally, Spirituality (0.296 and 0.293) emerged as the most influential contributor to the meaning of life for both high school and college students, followed by Zest (0.200 and 0.208) and the Love of learning (0.112 and 0.140). The results of the same analysis conducted among adults in Study 1 are presented on the right-hand side of the table. For subjective happiness, the explanatory rate was 39.6%, with the contributing strengths being Hope (0.244), Self-regulation (0.170), and Zest (0.102), in that order. Regarding meaning in life, the explanatory rate was 44.1%, with Spirituality (0.318) and Zest (0.286) emerging as the contributing strengths.

Overall, the findings from Study 2 suggest that the CST24 scores can be employed as measures in different populations, such as high school and college students. The results further validate the relationship between these scores and subjective happiness and meaning in life, as established in previous research, while also demonstrating a sufficiently high explanatory power. Notably, certain strengths, such as hope, zest, and spirituality, which have been recognized as core strengths in prior studies, play significant roles among the strengths assessed. The six-factor model demonstrated the best fit for the structure of strengths, consistent with the findings from the adult data in Study 1. However, other models also exhibited a certain degree of fit. Considering these outcomes, it is advisable to select the appropriate model based on the specific context in which the CST24 is applied, rather than aiming to reduce the number of strengths to enhance model fit.

## Study 3

In Study 3, the primary aim was to reconfirm the factor structure of the Character Strengths Test 24 (CST24) in the working adult population and examine the utility of the CST24 by assessing its relationship with various psychological indices. Multiple regression analysis was conducted to determine which strengths contribute to value-orientation, psychological well-being, morality foundation, sense of mission, and work-related psychological indicators among working adults.

## Methods

### Participants

A total of 564 Japanese working adults (283 males and 281 females) registered with an Online survey agency participated in the study. The mean age was 45.6 ± 13.0 (ranging from 23 to 64) years. The participants represented a range of ages, with each age group and gender roughly equally represented. The majority of participants (71.3%) were employed by companies or held company director positions. About 57.3% had general employee positions, and 62.2% worked for domestic Japanese corporations.

### Procedure

An Online survey agency facilitated the survey administration among Japanese working adults. The purpose and informed consent of the study were presented on-screen, and those who agreed to participate completed the survey. Data were collected from participants across different age groups and genders, ensuring adequate representation.

### Measures

In addition to the CST24, five scales related to well-being, ethical foundation and job fulfillment were employed. These scales include Value Orientation, Psychological Well-being, Moral Foundation Questionnaire, Expanded Sense of Mission Scale, and Organizational Engagement Scale. These scales used in Study 3 were developed and standardized in Japanese and have been validated and reliable in Japanese adults.

#### Value orientation [25, 26]

This scale measures value-intending orientation based on six types of values proposed by Spranger [25]. The six types are a) Rational orientation, b) Economy orientation, c) Aesthetic orientation, d) Religion orientation, e) Social orientation, and f) Power orientation.

#### Psychological well-being [27, 28]

This scale, originally proposed by Ryff & Keys [27], measures six dimensions of psychological well-being: a) Personal growth, b) Purpose in life, c) Autonomy, d) Self-acceptance, e) Environmental mastery, and f) Positive relationships with others.

#### Moral foundation questionnaire [29, 30]

This questionnaire assesses human moral judgments based on five inherent criteria: a) Care, b) Fairness, c) Loyalty, d) Authority, and e) Sanctity/Purity.

#### Expanded sense of mission Scale [31]

This scale measures the expanded sense of mission across four factors: a) Contribution to society, b) Contribution to colleagues, c) Upskilling-oriented nature of work, and d) Directions for developing new businesses.

#### Organizational engagement questionnaire [32]

This questionnaire comprises 43 items and measures ten subscales related to organizational engagement: a) Work fulfillment, b) Love for the organization, c) Teamwork, d) Trust in colleagues, e) Reliable supervisor, f) Trust in supervisor, g) Autonomy and feedback, h) Fairness of evaluation, i) Openness in the organization, and j) Recommendation behavior.

## Results and discussion

### Confirmatory factor analysis

Table 8 presents the results of the confirmatory factor analysis for the CST24 in the Japanese working adult population. The comparative fit index (CFI) for the two-factor model was 0.841 and for the six-factor model was 0.879, which was similar to the findings in Study 1. The lower limit of the root mean square error of approximation (RMSEA) was in the 0.07 range for all models, and the standardized root mean square residual (SRMR) values were consistent with those in Study 1.

**Table 8:**
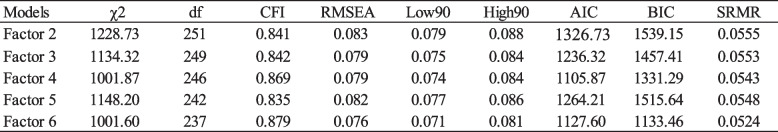
Confirmatory factor analysis model results with CST24 among working adults (*n* = 524) in Study 3

### Multiple regression analysis

Tables 9, 10, and 11 display the results of multiple regression analyses that examined the relationship between the CST24 strengths and various psychological indices. Table 9 focuses on value orientation and psychological well-being. The explanatory rates ranged from 40 to 50% for higher cases. It is noteworthy that Appreciation of beauty and excellence showed a notable positive contribution (coefficient = 0.414) to the subscale measuring aesthetic orientation in the value orientation measure. Similarly, Leadership demonstrated a significant positive contribution (coefficient = 0.447) to the subscale assessing power orientation in the same measure. In contrast, both of Spirituality (0.153) and Gratitude (0.219) showed significant contributions to the religion orientation. The social orientation was supported by multiple strengths, with Kindness (0.212) being a notable contributor. Personal growth was influenced by Curiosity (0.220) and Love of learning (0.210), while Love (0.160 and 0.213) played a role in positive relationships and environmental mastery.

**Table 9:**
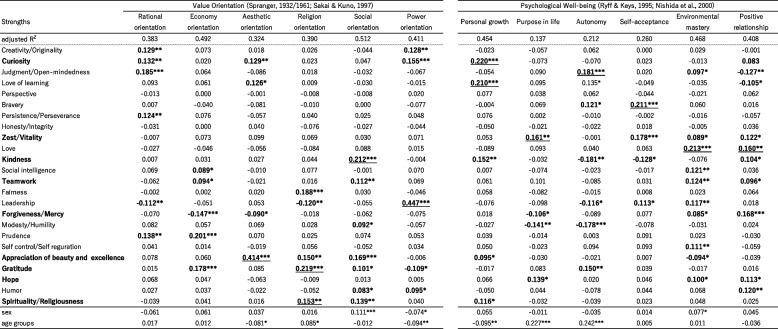
Results of multiple linear regression of Value orientation and Psychological well-being by CST24

^*^p < .05, ** *p* < .01, ***p < .001

**Table 10:**
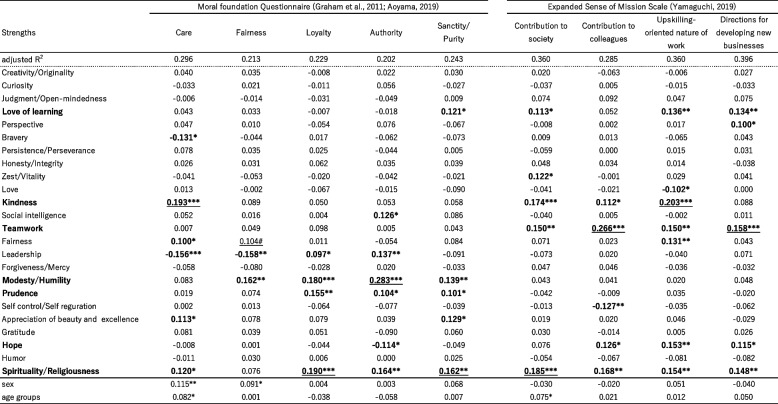
Results of Multiple linear regression of Moral foundation and Sense of mission scales by CST24

^*^p < .05, ** *p* < .01, ***p < .001, and #*p* = 054

**Table 11:**
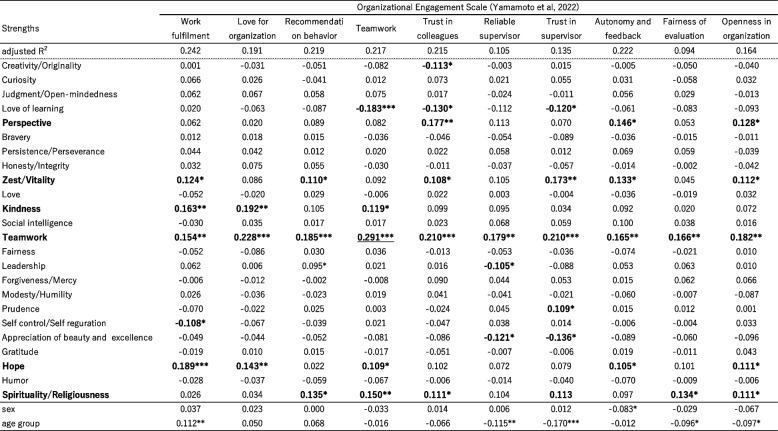
Results of Multiple linear regression of Organization engagement scales and ME-Work by CST24

^*^p < .05, ** *p* < .01, ***p < .001

Table 10 presents the results of the multiple regression analysis for moral foundation and sense of mission indices. The overall explanatory rates by strengths were in the 20% to 30% range, with Modesty showing significant contributions to moral foundation's Authority (0.283) and Care (0.193). Loyalty was influenced by Modesty (0.180) and Spirituality (0.190). However, Fairness did not contribute significantly to Moral Fairness. In terms of mission, Spirituality, Teamwork, Hope, Kindness, and Love of learning were the main contributors, indicating a limited number of strengths playing a significant role.

Table 11 presents the outcomes of the multiple regression analysis for the 10 measures of organizational engagement scale. It should be noted that the explanatory rates were relatively lower, indicating that the impact of strengths on these measures was less pronounced. However, Teamwork consistently exhibited a positive contribution across all indices, ranging from 0.165 to 0.291, while Perspective, Zest, Kindness, Hope, and Spirituality demonstrated some influence across different indicators. Surprisingly, Love of learning displayed a negative contribution to some engagement indicators, suggesting that lower levels of Love of learning were associated with those outcomes, contrary to the previous correlation analysis results.

Regarding Table 9, the explanatory rates for most of the subscales (Value orientation and Psychological well-being) were relatively high (from 0.4 to 0.5), while the variances for each of the scales in Tables 10 and 11 were relatively small. The lowest variance observed was 0.094 for the fairness of evaluation in Table 11, while most of the sense of mission scales had variances exceeding 0.3. These findings indicate that the larger variances of Value orientation and Wellbeing exhibited stronger associations with character strengths when compared to the Organization engagement scales.

In conclusion, the results of the multiple regression analysis unveiled that character strengths accounted for a significant portion of the variance in various psychological indices among working adults. Additionally, it became evident that the specific strengths contributing to the outcomes varied depending on the measure considered, emphasizing the importance of considering different strengths in different contexts.

## General discussion

### Validity and utility of CST24

The response distribution in the four samples indicates that the measurement conditions are met. Despite using a 7-point Likert scale, all 24 strength scores showed positive associations with subjective happiness, meaning in life, knowledge, and utilization of strengths. The scale also demonstrated convergent and discriminant validity by exhibiting positive correlations with positive self-compassion and negative correlations with negative self-compassion. Moreover, the retest reliability over a 30-day interval was high, suggesting that the CST24 can be used as a shortened version of the strengths scale to assess the 24 strengths.

Compared to the Comprehensive Strengths Rating Form (CSRF), the CST24 has the advantage of being a shortened version with single-sentence questions, making it more convenient. While the CSRF demonstrated criterion-related validity using the VIA Inventory of Strengths (VIA-IS), which is superior to the CST24 in this aspect, the criterion-related validity of the CST24 should be confirmed in future research. It would be feasible to conduct a study using both the CSRF and the CST24 simultaneously since a Japanese version of the CSRF has recently been developed [33].

However, it is important to note that the CST24, which evaluates 24 strengths in a single item, is naturally less precise than the 240-item VIA-IS. Therefore, it cannot be considered an alternative measure to the VIA-IS or other large-scale assessments. Nevertheless, the brief scale can still be useful and convenient for longitudinal and exploratory purposes, as demonstrated in Study 3.

The structure of character strengths has been extensively studied, with previous research proposing two- to six-factor models. In this study, we examined these factor models using confirmatory factor analysis and found that they showed good structure, particularly the six-factor model, which exhibited a sufficient goodness of fit for high school students. Although the goodness of fit for the other samples was not as high, the six-factor model still had the best fit, indicating the factorial validity of the CST24, consistent with previous attempts to propose a strength structure.

Regarding the classification of strengths into virtues, Peterson and Seligman proposed six virtues without specifying the criteria for this classification, unlike the 10 criteria used for selecting the 24 strengths. The CSV handbook also does not provide total scores for each virtue due to the criterion stating that each strength is morally valued in its own right. Additionally, it suggests that strengths are unidimensional and cannot be decomposed into other strengths in the classification. Therefore, it is inappropriate to delete strengths based on factor analysis results. This aligns with the arguments made by Rush and Proyer [34], who caution against removing or adding strengths based on factor analysis results.

The future direction of research should include clarifying the roles of different character strengths, understanding the functions of general core strengths, strengths that contribute strongly to specific positive psychological concepts, and those that contribute less to such outcomes as happiness and meaning in life. Additionally, research is needed to explore the synergy, complementary effects, additive effects, or anergy effects of multiple strengths, as indicated by the negative contributions observed. Furthermore, accumulating evidence on effective combinations of strengths for achieving positive outcomes in practical interventions is crucial, considering the multifaceted feature of such interventions [35].

In conclusion, the development of the 24-item Character Strengths Test (CST24) in Japanese and its validation and retest reliability have been examined in this study. The results demonstrate internal consistency, retest reliability, and a factor structure that aligns with previous findings using the VIA-IS. The strengths scores show positive associations with subjective well-being, presence of meaning in life, knowledge of strengths, and their utilization. The CST24 is a valid instrument that can serve as a brief scale for assessing 24 different strengths in separate sample population in Study 2. Additional evidence of its validity has been provided in Studies 3, where different roles and associations of strengths were explored. Future research should address the limitations mentioned and further investigate the topics outlined for a better understanding of character strengths.

### Limitations

The main limitation of the CST24 is the absence of an association with the VIA-IS, which is considered a standard measure of character strengths. While it would be desirable to establish criterion-related validity using the VIA-IS, there is limited access to VIA data, including the Japanese version. However, given the recent development of the Japanese version of the CSRF [33], conducting a correlation study between the CST24 and the Japanese CSRF is a promising option. On the positive side, all items of the CST24 are publicly available, enabling researchers to assess their content validity and make necessary improvements.

### Supplementary Information

**Additional file 1.**

## Data Availability

The Japanese version of the questionnaire employed in this study is provided as supplementary material, facilitating its utilization for future investigations. The dataset produced and examined in the present research will be accessible upon reasonable request from the corresponding author (SS).

## Abbreviations

BST: Brief Strengths Test
CSRF: Character Strengths Rating Form
CST24: Character Strengths Test 24
CSV: Character Strengths and Virtues
GAT: Global Assessment Tool
MLQ-p: Meaning in Life Questionnaire—Presence of meaning score
MLQ-s: Meaning in Life Questionnaire – Search for meaning score
RMSEA: The root mean square error of approximation
SCS: Self-Compassion Scale
SHS: Subjective Happiness Scale
SKS: Strength Knowledge Scale
SRMR: The standardized root mean square residual
SS: Satoshi Shimai
SUS: Strength Use Scale
VIA-IS: The Values-in-Action, Inventory of Strengths
YU: Yu Urata
