# Supplementary material for: Development and validation of the Character Strengths Test 24 (CST24): a brief measure of 24 character strengths
Source: BMC Psychol. 2023 Aug 18;11:238. doi: 10.1186/s40359-023-01280-6 (PMC10436548; doi:10.1186/s40359-023-01280-6)
Supplement: Supplementary file 1 — Additional file 1. [file 40359_2023_1280_MOESM1_ESM.docx]

Supplementary material: CST24 Japanese questionnaire

人間には、ひとによって、さまざまな良いところ(強み)があります。以下の強みの説明の文章を読んで、自分にどのくらいそういうところがあるかを考えてその程度を回答してください。

|  | まったくあてはまらない | ほとんどあてはまらない | あまりあてはまらない | どちらともいえない | 少しあてはまる | だいたいあてはまる | 非常によくあてはまる |
| --- | --- | --- | --- | --- | --- | --- | --- |
| 1. 独創性：わたしは、新しい見方や考え方を思いつき、独自の方法で解決につなげます | １ | ２ | ３ | ４ | ５ | ６ | ７ |
| 1. 好奇心：わたしは、新しいものが好きで、新しい人と出会ったり、新しい経験をしたいと思っています | １ | ２ | ３ | ４ | ５ | ６ | ７ |
| 1. 判断力：わたしは、ものごとをいろいろな側面から検討し、よく吟味した根拠をもって結論を下します | １ | ２ | ３ | ４ | ５ | ６ | ７ |
| 1. 向学心：わたしは、自分の知識や経験を深めたいと考えて、新しいことを学ぼうと熱心に努力します | １ | ２ | ３ | ４ | ５ | ６ | ７ |
| 1. 見通し：わたしは、ものごとのの流れや大筋をよくとらえていて、他の人から相談されることも多いです | １ | ２ | ３ | ４ | ５ | ６ | ７ |
| 1. 勇気：わたしは、さまざまな困難を真正面からとらえ、怖がったりしりごみしないで挑戦します | １ | ２ | ３ | ４ | ５ | ６ | ７ |
| 1. 勤勉性：わたしは、障害があったとしても、やり始めたことを完成するまでやり続けることができます | １ | ２ | ３ | ４ | ５ | ６ | ７ |
| 1. 正直：わたしは、まじめで信頼されており、どんなときにも嘘をつくことはありません | １ | ２ | ３ | ４ | ５ | ６ | ７ |
| 1. 熱意：わたしは、人生と日常生活に熱心で、いつも全力でエネルギッシュに活動します | １ | ２ | ３ | ４ | ５ | ６ | ７ |
| 1. 親密性：わたしは、温かくて他の人に寄り添うことのでき、他の人からも好かれています | １ | ２ | ３ | ４ | ５ | ６ | ７ |
| 1. 親切心：わたしは、他の人の面倒をみてあげて、何かしてあげたいという気持ちに満ち溢れています | １ | ２ | ３ | ４ | ５ | ６ | ７ |
| 1. 社会的知能：わたしは、その場の流れや人の気持ちによく気がつき、先回りして行動することができます | １ | ２ | ３ | ４ | ５ | ６ | ７ |
| 1. 忠誠心：わたしは、グループのメンバーと協力して、チームのために働き、積極的に責任を果たします | １ | ２ | ３ | ４ | ５ | ６ | ７ |
| 1. 公平性：わたしは、平等に機会があることが大切だと思い、みんなに同じように接します | １ | ２ | ３ | ４ | ５ | ６ | ７ |
| 1. リーダーシップ：わたしは、誰かに従うより、自分がリ－ダーとしてみんなのために働くのが得意です | １ | ２ | ３ | ４ | ５ | ６ | ７ |
| 1. 寛容性：わたしは、理不尽な扱いを受け流すことができ、他人の失敗を許すことができます | １ | ２ | ３ | ４ | ５ | ６ | ７ |
| 1. 謙虚：わたしは、自分の足りないところを認め、自分よりも他の人の成功を喜ぶほうです | １ | ２ | ３ | ４ | ５ | ６ | ７ |
| 1. 思慮深さ：わたしは、あとで後悔しないように、慎重に計画し、十分に注意深く準備します | １ | ２ | ３ | ４ | ５ | ６ | ７ |
| 1. 自己制御：わたしは、とても自制心があり、自分の感情や行動をコントロールして、平静で落ち着いています | １ | ２ | ３ | ４ | ５ | ６ | ７ |
| 1. 審美心：わたしは、美しいものや素晴らしいものを見つけて、それに心打たれて感激することが多いです | １ | ２ | ３ | ４ | ５ | ６ | ７ |
| 1. 感謝心：わたしは、人生の良い出来事を当たり前とは思わず、ありがたく感じその気持ちを伝えます | １ | ２ | ３ | ４ | ５ | ６ | ７ |
| 1. 希望：わたしは、望みがかなうことを期待し、それを信じて楽しく励むことができます | １ | ２ | ３ | ４ | ５ | ６ | ７ |
| 1. ユーモア：わたしは、人を笑わせるのが好きで、落ち込んだ雰囲気をなごませて楽しくすることができます | １ | ２ | ３ | ４ | ５ | ６ | ７ |
| 1. 精神性：わたしは、人生には大切な意味があると信じており、それに従って行動します | １ | ２ | ３ | ４ | ５ | ６ | ７ |
